# Prediction of Ki-67 expression in hepatocellular carcinoma: a dual-center study based on T2-weighted imaging habitat analysis

**DOI:** 10.2478/raon-2026-0032

**Published:** 2026-06-26

**Authors:** Xiaojun Zheng, Lihong Huang, Mengjie Huang, Bin Yu, Shiji Qin, Deyou Huang

**Affiliations:** 1 Graduate School, Youjiang Medical University for Nationalities, Baise, China; 2 Department of Radiology, The Affiliated Hospital of Youjiang Medical University for Nationalities, Baise, China

**Keywords:** hepatocellular carcinoma, habitat imaging, radiomics, prognostic prediction

## Abstract

**Background:**

Limited research has applied habitat imaging to evaluate the association between T2-weighted magnetic resonance imaging (T2WI-MRI) features and Ki-67 expression in hepatocellular carcinoma HCC). This study aimed to link T2WI habitat-derived parameters to Ki-67 status and aggressiveness in HCC.

**Patients and methods:**

This dual-center retrospective study, enrolled patients with pathologically confirmed HCC undergoing preoperative MRI (2020–2024). Using Ki-67 index cutoff (20%). Tumor habitat partitioning was performed using k-means clustering (k = 5), followed by extraction of both habitat-specific radiomic features and conventional whole-tumor features. Feature selection was conducted using least absolute shrinkage and selection operator (LASSO) regression with 10-fold cross-validation. Three predictive models were constructed with the ExtraTrees algorithm: a habitat model, a radiomics model, and a clinical model. Model performance was assessed using the area under the receiver operating characteristic curve (AUC), DeLong test, and decision curve analysis (DCA).

**Results:**

T2WI-based habitat imaging enables a noninvasive assessment of intratumoral heterogeneity, significantly improving the prediction of Ki-67 expression status in HCC. This approach may provide a promising imaging biomarker for molecular subtyping and support personalized preoperative treatment strategies.

**Conclusions:**

T2WI habitat imaging enables improved Ki-67 prediction, supporting informed therapeutic decisions in HCC.

## Introduction

Hepatocellular carcinoma (HCC), ranking as the sixth most common malignancy globally with approximately 830,000 new cases annually, presents significant clinical challenges due to its substantial heterogeneity and aggressive biological behavior.^1,2^ The Ki-67 proliferation index, established as a robust histopathological marker of cellular proliferation, demonstrates strong correlation with tumor invasiveness and postoperative survival outcomes in HCC patients.^3-5^ Elevated Ki-67 expression consistently portends unfavorable prognosis, underscoring its clinical relevance for guiding treatment stratification and prognostic evaluation. However, current assessment remains dependent on postoperative pathological evaluation, with percutaneous biopsy limited by sampling variability and inability to serially monitor treatment response.^6,7^ Consequently, developing a reliable noninvasive method for preoperative Ki-67 status prediction represents a crucial unmet need in HCC management-it would alter clinical practice by guiding preoperative risk stratification, individualizing treatment selection (e.g., radical surgery vs. neoadjuvant therapy), and facilitating noninvasive serial response monitoring, thereby overcoming key limitations of invasive assessments.^8^

While computed tomography CT and magnetic resonance imaging(MRI) are well-established for HCC diagnosis and staging, their capacity to characterize intratumoral heterogeneity remains suboptimal.^9-11^ Conventional radiomics approaches based on whole-tumor analysis often fail to capture the spatially heterogeneous “mosaic” architecture characteristic of HCC.^12^ Pathological evidence confirms that Ki-67 high-expression regions frequently demonstrate clustered distribution patterns, particularly at the tumor periphery, with close spatial correlation to areas of enhanced microvascular density.^13^,^14^ Although diffusion-weighted imaging (DWI) has shown preliminary promise for Ki-67 assessment, technical limitations including susceptibility artifacts and signal attenuation constrain its reproducibility.^15^

T2-weighted Imaging (T2WI) MRI provides exceptional sensitivity to tissue water content variations, offering unique capabilities for noninvasive characterization of intratumoral heterogeneity including necrosis, fibrosis, and cellularity. Habitat imaging methodology, employing unsupervised clustering algorithms such as k-means, enables segmentation of tumors into biologically distinct subregions and has demonstrated substantial success in glioma molecular classification.^16^ Nevertheless, the relationship between T2WI-derived habitat characteristics and HCC proliferative activity spatial patterns remains inadequately explored, particularly in the context of multifocal HCC within cirrhotic livers where interlesional heterogeneity may confound predictive accuracy.^17^

This investigation aimed to develop and validate a T2WI-based habitat imaging model for non-invasive prediction of Ki-67 expression in HCC, with particular focus on its potential clinical applications for risk stratification and treatment guidance.

## Patients and methods

### Study population

This retrospective study was approved by the Ethics Committees of the Affiliated Hospital of Youjiang Medical University for Nationalities and the Affiliated Southwest Hospital of Youjiang Medical University for Nationalities (Approval number: 2025091701). Given the use of anonymized retrospective data, the requirement for informed consent was waived in accordance with national guidelines on biomedical research ethics.

Patients with pathologically confirmed HCC who underwent surgical resection at the two participating centers between January 2020 and January 2024 were eligible. Inclusion criteria were: (1) solitary, resectable HCC demonstrated on imaging; (2) no prior HCC-related treatment before baseline MRI; (3) liver MRI including T2WI performed within 30 days before surgery with sufficient image quality for analysis; and (4) availability of complete demographic, laboratory, and pathological data including standardized Ki-67 proliferation index. Exclusion criteria included: (1) unresectable or multifocal HCC; (2) prior treatment before baseline MRI; (3) images with significant artifacts or insufficient anatomic clarity precluding tumor delineation, confirmed by two senior radiologists; and (4) incomplete clinical or pathological information.

**FIGURE 1.**
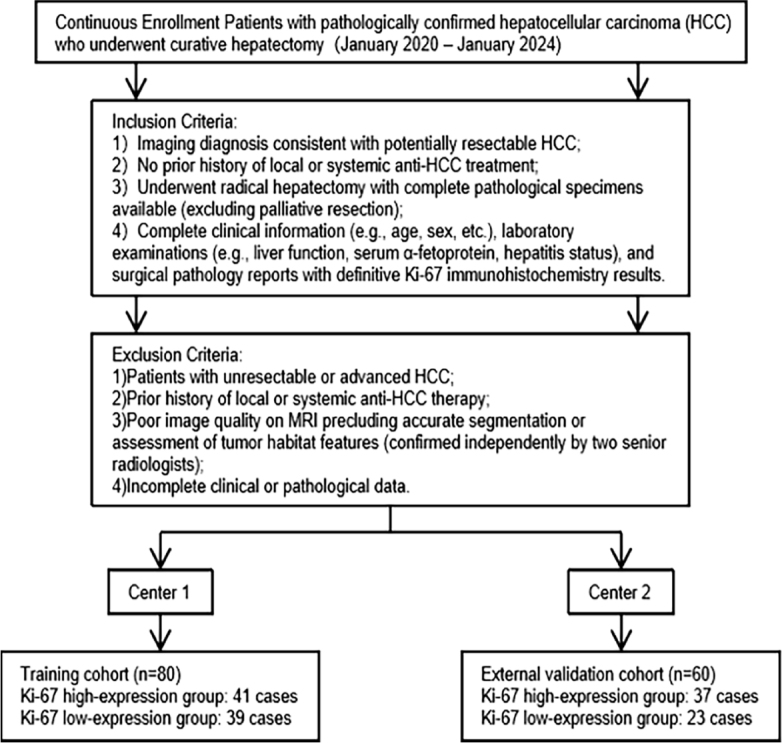
Patient inclusion and exclusion flowchart for the hepatocellular carcinoma (HCC) dual-center study cohort.

A total of 140 patients met the criteria, comprising 80 from the Affiliated Hospital (Center 1, training cohort) and 60 from the Affiliated Southwest Hospital (Center 2, external validation cohort). Based on immunohistochemical Ki-67 labeling index, patients were stratified into high-expression (≥ 20%) and low-expression (< 20%) groups (Figure 1).^18^

### Clinical data collection and follow-up

Demographic, laboratory, and histopathological data were retrieved from electronic medical records. Variables included: age, sex, hepatitis B surface antigen (HBsAg), alpha-fetoprotein (AFP), albumin (ALB), aspartate transaminase (AST), alanine aminotransferase (ALT), platelet count (PLT), prothrombin time (PT), and Ki-67 proliferation index.

Postoperative surveillance consisted of imaging (ultrasound, CT, or MRI) every 3–6 months and laboratory testing (AFP, liver function). Tumor recurrence was defined as the imaging detection of new intrahepatic or extrahepatic lesions. Followup was censored at recurrence or the last clinical visit (May 2025).

### Immunohistochemistry for Ki-67

All resected specimens underwent hematoxylineosin staining and Ki-67 immunohistochemistry using a mouse anti-human Ki-67 monoclonal antibody, according to manufacturer protocols. Two pathologists (≥ 10 years of experience) independently and blindly evaluated all slides. For each case, 10 random high-power fields (400×) were examined, and the labeling index was calculated as the percentage of positively stained tumor nuclei. Ki-67 expression was classified as high (≥ 20%) or low (< 20%). To justify the 20% Ki-67 cutoff value used in this study, we primarily based our selection on three key considerations: first, this cutoff is consistent with the most widely accepted standards in existing HCC-related studies, which have frequently adopted 20% as the threshold to distinguish high and low Ki-67 expression levels for evaluating tumor proliferation activity and predicting patient prognosis (consistent with the references cited in our manuscript). Second, the 20% cutoff has been validated in multiple large-cohort studies focusing on HCC, demonstrating good discriminatory ability in distinguishing tumors with different proliferative potentials and clinical outcomes. Third, we also referred to the clinical practice guidelines for HCC diagnosis and treatment, which recommend 20% as a reasonable threshold for Ki-67 expression classification when assessing tumor aggressiveness and guiding individualized treatment strategies. Collectively, these considerations confirm that the 20% Ki-67 cutoff used in our study is scientifically reasonable, clinically relevant, and consistent with current academic consensus and clinical practice.^19-21^

### MRI acquisition, preprocessing, and tumor segmentation

All patients underwent preoperative liver MRI. Examinations were performed on 3.0T (GE Discovery MR 750, Siemens Skyra) and 1.5T scanners (Siemens Amira, GE SIGNA Creator, Canon Vantage Elan), using standardized protocols. Sequences included T1-weighted T1WI in-phase and out-of-phase imaging, fast recovery fast spinecho T2WI, and DWI. Respiratory gating was applied to minimize motion artifacts. Detailed parameters are provided in Supplementary Table 1. Images were exported in DICOM format.

Preprocessing included N4 bias field correction to address inter-scanner field inhomogeneity, resampling to isotropic voxels (1 × 1 × 1 mm^3^), and normalization of window level/width (6236/2774).

**FIGURE 2.**
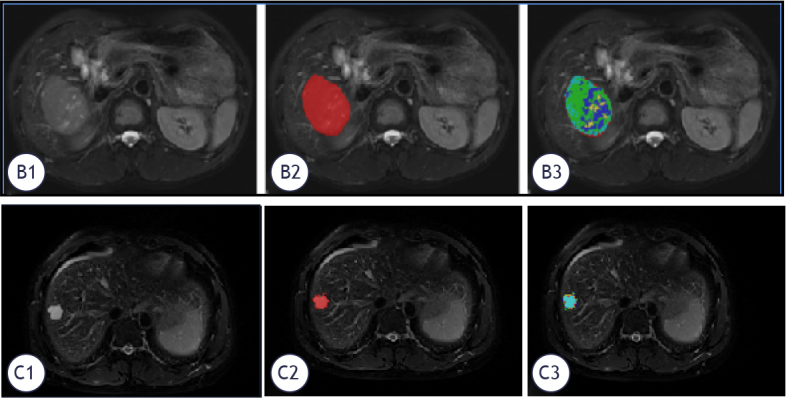
T2WI-based habitat segmentation of hepatocellular carcinoma (HCC) and its correlation with Ki-67 expression status. **(B1-B3)**: HCC with Ki-67 -high expression (20%); **(C1-C3)**: HCC with Ki-67 low expression (10%). Representative T2WI images and corresponding habitat segmentation of HCC with different Ki-67 expression levels. The red area indicates the tumor high-proliferation potential region (correlated with high Ki-67 expression), and the blue area indicates the low-proliferation potential region (correlated with low Ki-67 expression).

Tumor segmentation was performed manually on T2WI using ITK-SNAP (v3.8, www.itksnap.org) by a radiologist with > 5 years of experience, delineating the tumor layer by layer in axial slices. Lesions with maximum axial diameter < 0.5 cm were excluded to minimize volume effects. Contrast-enhanced T1WI and DWI were referenced to aid tumor-arenchyma differentiation. Final contours were reviewed by a second radiologist (> 10 years of experience), yielding the tumor volume of interest (VOI). To address the reviewer’s valuable suggestion and assess the reliability and reproducibility of tumor segmentation, we performed inter- and intra-observer segmentation variability analysis. For intra-observer variability, the radiologist with > 5 years of experience repeated the segmentation of 30 randomly selected HCC lesions after a 2-week interval, with no access to the original segmentation results. For inter-observer variability, a third independent radiologist (with 6–8 years of experience) who was unaware of the study design and patient outcomes performed segmentation on the same 30 randomly selected lesions following the same standard. The consistency of the segmentation results was carefully evaluated and compared between the repeated segmentations (intra-observer) and between different radiologists (inter-observer), which confirmed the robustness and reproducibility of our tumor segmentation protocol Figure 2).

### Radiomics feature extraction

Using the PyRadiomics package (https://github.com/Radiomics/pyradiomics), a total of 1,834 features were extracted from each VOI on T2WI images, including:

14 shape features,

360 first-order features,

280 gray-level dependence matrix (GLDM) features,

320 gray-level size zone matrix (GLSZM) features,

100 neighborhood gray-tone difference matrix (NGTDM) features,

320 gray-level run length matrix (GLRLM) features,

440 gray-level co-occurrence matrix (GLCM) features.

### Habitat analysis

Voxel-level tumor heterogeneity was explored using the K-means clustering algorithm (scikit-learn, https://github.com/scikit-learn/scikit-learn). Clustering was based on T2WI signal intensity, with the number of clusters (k) varied from 2 to 5. The optimal k was selected using the Calinski-Harabasz index, which peaked at k = 5, yielding the final tumor habitat partition.^22^

Cluster maps were visualized with distinct colors representing each subregion. Radiomics features were then extracted from each subregion, producing 9,170 habitat features (1,834 × 5). To mitigate redundancy caused by overlapping cluster centers, cross-cluster feature averages were calculated to generate the representative habitat feature set.

### Feature selection and model construction

A multi-stage strategy was implemented to optimize model stability and prevent overfitting. All features were Z-score normalized. Initial screening was performed with univariate tests (t-test or Mann-Whitney U, P < 0.05). Features with Pearson correlation coefficients > 0.9 were excluded to reduce collinearity. The least absolute shrinkage and selection operator (LASSO) with 10-fold cross-validation was subsequently applied for final selection.

Three predictive models were constructed using the ExtraTrees algorithm: a radiomics model (whole-tumor features), a habitat model (subregion features), and a clinical model (demographic and laboratory variables). To address class imbalance, synthetic minority oversampling (SMOTE) was applied. Hyperparameters were tuned using grid search with 5-fold cross-validation. Model interpretability was evaluated using Shapley additive explanation (SHAP) values to quantify feature contributions.

### Statistical analysis

All analyses were performed using Python 3.7.12. Statistical tests were implemented with Statsmodels (v0.13.2), and machine learning algorithms with Scikit-learn (v1.0.2). Continuous variables were compared with independent-samples t-test (normal distribution) or Mann-Whitney U test (non-normal distribution). Model discrimination was assessed by the area under the receiver operating characteristic curve (AUC). Clinical utility was evaluated using decision curve analysis (DCA).

## Results

### Baseline characteristics of the study population and clinical model construction results

In the training cohort of HCC patients, the Ki-67 high-expression group comprised 41 cases (32 males, 9 females; age 54.44 ± 11.45 years), while the Ki-67 low-expression group included 39 cases (29 males, 10 females; age 54.03 ± 11.45 years). In the external validation cohort, the Ki-67 high-expression group comprised 37 HCC patients (31 males, 6 females; age 53.76 ± 11.91 years), while the Ki-67 low-expression group included 23 patients (19 males, 4 females; age 55.17 ± 12.80 years). In the training set, HCC patients in the Ki-67 high-expression group exhibited significantly higher AFP levels than those in the low-expression group (P < 0.05), making AFP the sole predictor selected for the clinical model. No statistically significant differences were observed between the training and external validation sets regarding age, gender, HBsAg, ALB, AST, ALT, PLT, or PT (all P > 0.05) (Table 1). In the clinical model constructed by ExtraTrees, the training set achieved accuracy, AUC, sensitivity, and specificity of 0.750, 0.775, 0.854, and 0.641, respectively. The external validation set achieved accuracy, AUC, sensitivity, and specificity of 0.617, 0.615, 0.649, and 0.565, respectively.

**TABLE 1.** Comparative analysis of clinical baseline data across hepatocellular carcinoma patient datasets

Clinical Data	Training Cohort		P-value	External Validation Cohort		P-value
	Ki-67 low-expression group	Ki-67 high-expression group		Ki-67 low-expression group	Ki-67 high-expression group	
**Age** **(years old $\bar{\text{x}}$ ± s)**	54.03±11.45	54.44±11.60	0.873	55.17±12.80	53.76±11.91	0.665
**Sex** **(number of cases, (%))**			0.901			1.0
**Male**	29 (74.36)	32 (78.05)		19 (82.61)	31 (83.78)	
**Female**	10 (25.64)	9 (21.95)		4 (17.39)	6 (16.22)	
**HBsAg**	1006.1±266.41	1108.43±910.28	0.209	404.61±774.98	670.74±1560.00	0.745
**AFP**	237.50±456.96	547.53±516.66	0.001	273.98±562.51	4128.08±17293.40	0.073
**ALB**	37.62±4.42	38.16±3.60	0.55	39.75±4.34	39.11±4.92	0.61
**AST**	57.38±60.05	56.68±59.60	0.889	91.83±139.89	51.43±56.38	0.879
**ALT**	57.52±81.88	58.88±72.24	0.321	103.63±161.34	69.98±91.43	0.749
**PLT**	210.00±98.92	207.90±91.08	0.806	183.37±76.63	202.96±84.05	0.648
**PT**	11.99±1.35	12.29±2.55	0.942	12.79±1.42	12.67±1.27	0.749

1 AFP = alpha-fetoprotein; ALB = albumin; ALT= alanine aminotransferase; AST = aspartate aminotransferase; HBsAg = hepatitis B surface antigen; PLT = platelet count; PT = prothrombin time

### Habitat imaging model results

After t/Mann-Whitney U tests, Pearson correlation analysis, and Lasso regression dimensionality reduction, 10 radiomics features were finally selected for predictive model construction. In the habitat imaging model constructed using ExtraTrees, the training set achieved accuracy, AUC, sensitivity, and specificity of 0.938, 0.984, 0.976, and 0.897, respectively. The external validation set achieved accuracy, AUC, sensitivity, and specificity of 0.850, 0.910, 0.919, and 0.739, respectively.

### Imagingomics model results

After t/Mann-Whitney U tests, Pearson correlation analysis, and Lasso regression dimension reduction, nine radiomics features were finally selected for model construction. In the radiomics model constructed using ExtraTrees, the training set accuracy, AUC, sensitivity, and specificity were 0.713, 0.814, 0.561, and 0.872, respectively. For the external validation set, the accuracy, AUC, sensitivity, and specificity were 0.750, 0.746, 0.757, and 0.739, respectively.

### Comparison of model performance

**FIGURE 3.**
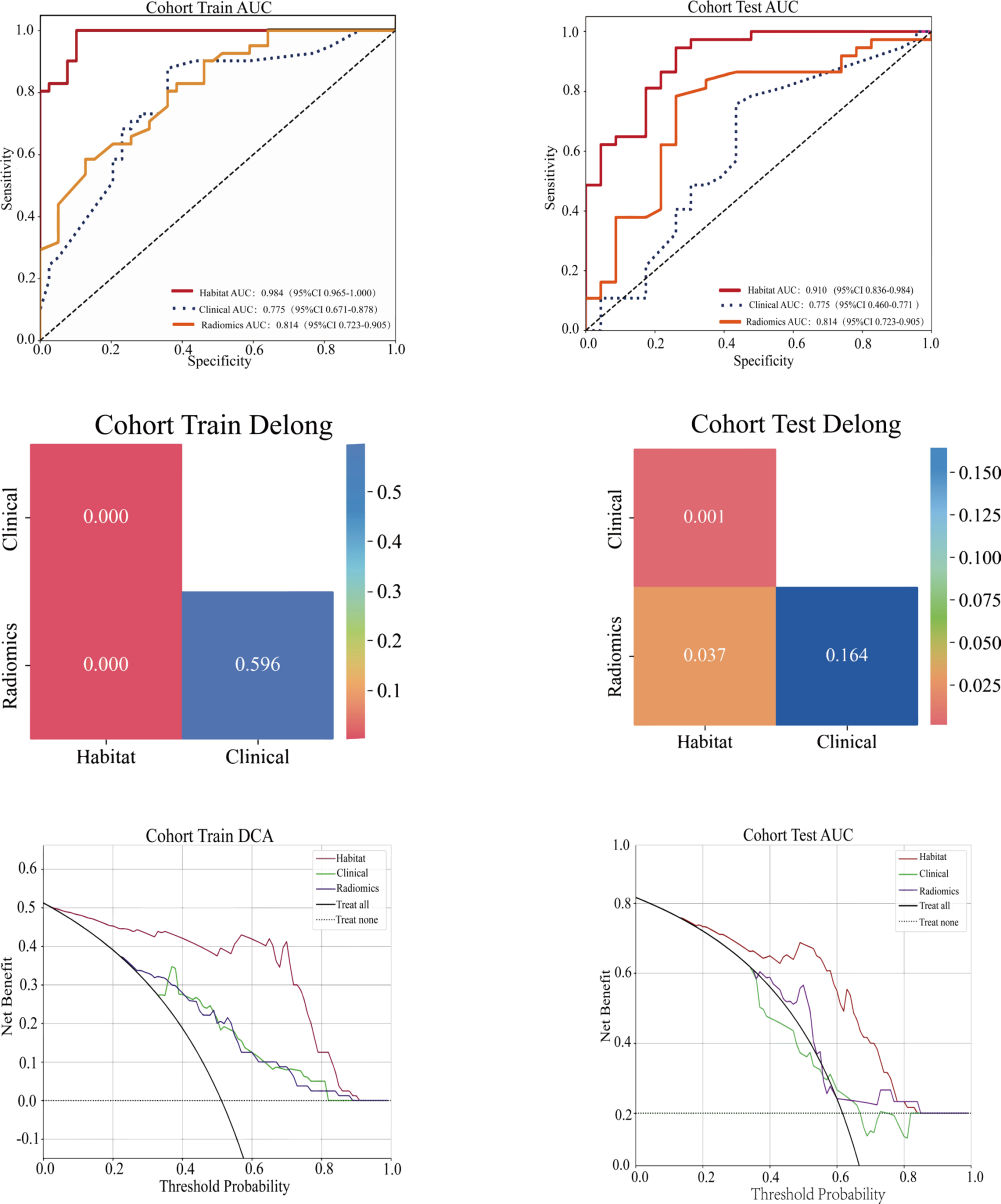
ROC curves, DeLong test results and decision curve analysis (DCA) for evaluating the predictive performance of three models in predicting HCC Ki-67 expression. Receiver operating characteristic (ROC) curves showing the diagnostic performance of the habitat, radiomics and clinical models in the training and external validation cohorts, with key area under the curve (AUC) values labeled. DeLong test results verifying the statistical significance of performance differences between models (P values labeled) in the training and external validation cohorts. Decision curve analysis (DCA) curves demonstrating the net clinical benefit of the three models in the training and external validation cohorts; the habitat model yielded the highest net benefit across all threshold probabilities, with notable clinical value when the threshold probability exceeded 0.2. The habitat model outperformed the radiomics and clinical models in all assessments (P<0.05).

As demonstrated by the receiver operating characteristic (ROC) curves and DeLong tests for both the training and external validation sets (Figure 3), the habitat imaging model exhibited significantly higher AUC values than both the radiomics model and clinical model in both datasets (P < 0.05). DCA indicated that the habitat imaging model demonstrated superior clinical net benefit over both the radiomics model and clinical model in both the training and external validation sets (Table 2).

**TABLE 2.** Predictive performance of various models for Ki-67 expression in hepatocellular carcinoma

Model	Cohort	Accuracy	Area under the curve	95% confidence interval	Sensitivity	Specificity
Habitat analysis	Training Cohort	0.938	0.984	0.9647-1.0000	0.976	0.897
	External Test Cohort	0.850	0.910	0.8355-0.9835	0.919	0.739
Radiomics	Training Cohort	0.713	0.814	0.7234-0.9045	0.561	0.872
	External Test Cohort	0.750	0.746	0.6102-0.8810	0.757	0.739
Clinical features	Training Cohort	0.750	0.775	0.6715-0.8782	0.854	0.641
	External Test Cohort	0.617	0.615	0.4597-0.7706	0.649	0.565

### SHAP analysis

**FIGURE 4.**
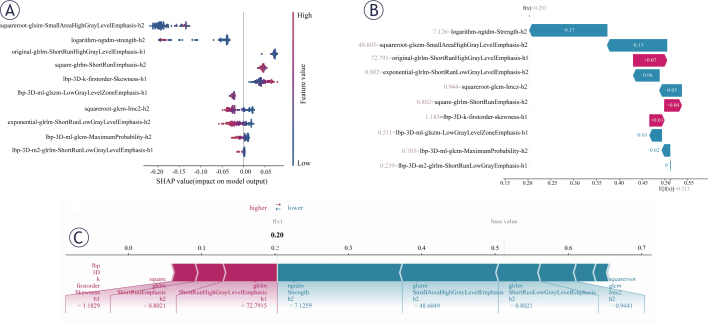
SHAP visualizations for model interpretability. **(A)** SHAP summary plot (beeswarm plot) displaying feature importance ranked by mean absolute SHAP values. Each point represents a single patient, with horizontal displacement indicating the magnitude and direction of the feature’s effect on model output. **(B)** SHAP waterfall plot illustrating how each feature’s contribution (SHAP value) accumulates from the base value to the final prediction for an individual case. Each bar represents the magnitude and direction (positive or negative) of a feature’s contribution to the prediction. **(C)** SHAP force plot demonstrating how features combine to push the model output from the base value (average prediction) to the final predicted value for a specific instance. Red arrows indicate features increasing the prediction, while blue arrows represent features decreasing the prediction, with arrow length corresponding to contribution strength.

SHAP values were further applied to analyze the contribution levels and influence directions of each habitat imaging feature within the top-performing habitat imaging model. Results indicate that square-root_glszm_SmallAreaHighGrayLevelEmphasis_h2, square_glrlm_ShortRunEmphasis_h2, lbp_3D_k_firstorder_Skewness_h1, and lbp_3D_m1_glszm_LowGrayLevelZoneEmphasis_h1 positively influenced the prediction of HCC Ki-67 high expression. Conversely, squareroot_glcm_Imc2_h2, original_glrlm_ ShortRunHighGrayLevelEmphasis_h1, lbp_3D_ m1_glcm_MaximumProbability_h2, exponential_glrlm_ShortRunLowGrayLevelEmphasis_h2, logarithm_ngtdm_Strength_h2, and lbp_3D_ m2_glrlm_ShortRunLowGrayLevelEmphasis_h1 exhibited negative effects. Among these, square-root_glszm_SmallAreaHighGrayLevelEmphasis_h2 contributes most significantly to the prediction model. Taking the first sample as an example, the waterfall plot and force diagram visualization reveal that logarithm_ ngtdm_Strength_h2, squareroot_glszm_SmallAreaHighGrayLevelEmphasis_h2, exponential_glrlm_ShortRunLowGrayLevelEmphasis_h2, squareroot_glcm_Imc2_h2, lbp_3D_m1_glszm_ LowGrayLevelZoneEmphasis_h1, lbp_3D_m1_ glcm_MaximumProbability_h2, and lbp_3D_ m2_glrlm_ShortRunLowGrayLevelEmphasis_h1 exerted negative regulatory effects in this sample prediction. Conversely, original_glrlm_ ShortRunHighGrayLevelEmphasis_h1, square_glrlm_ShortRunEmphasis_h2, and lbp_3D_k_first-order_Skewness_h1 exert positive regulation. The final predicted probability for this sample is 0.203, indicating a low risk of Ki-67 overexpression (Figure 4).

## Discussion

The T2WI-MRI habitat imaging model for predicting HCC Ki-67 expression levels holds profound oncologic implications, as it bridges the gap between imaging phenotypes and the molecular biology of tumor proliferation, thereby providing actionable insights for patient prognosis assessment and clinical decision-making. Among the 140 HCC patients enrolled in this study, the non-invasive Ki-67 prediction model constructed using T2WI-MRI habitat subregion features demonstrated outstanding discriminatory performance in both the training set (AUC = 0.984) and external validation set (AUC = 0.910), significantly outperforming radiomics models (training set AUC = 0.81; external validation set AUC = 0.75) and clinical models (training set AUC = 0.78; external validation set AUC = 0.62). Importantly, this superior performance is rooted in the unique ability of habitat imaging to dissect intratumoral heterogeneity—a key oncologic hallmark of HCC that dictates tumor aggressiveness, treatment resistance, and patient outcomes. Previous habitat imaging studies have predominantly focused on gliomas and breast cancer, with limited application in predicting HCC pathological features.^23-25^ This study extends bioimage analysis to HCC tumor microenvironment assessment, not only providing comprehensive supplementary information for evaluating patient prognosis but also uncovering spatial patterns of tumor proliferation that are tightly linked to HCC oncogenesis and progression. Maestri *et al*.^26^ further supports this approach, demonstrating significant associations between HCC tumor cell heterogeneity in spatial contexts and patient prognosis, which aligns with our findings and reinforces the oncologic value of habitat-based analysis.

Ki-67 is a non-histone protein expressed in the nuclei of proliferating cells (G1, S, G2, M phases). With its expression level typically assessed via immunohistochemical staining as the percentage of positive cells, directly reflecting cellular proliferation activity^27^, an essential oncologic marker that serves as a surrogate for tumor aggressiveness. In HCC, high Ki-67 expression indicates aggressive tumor behavior, poor differentiation, increased metastasis risk, and early postoperative recurrence, correlating significantly with poor patient prognosis^3^,^28^,^29^, thus, non-invasive prediction of Ki-67 status via habitat imaging addresses a critical oncologic unmet need by enabling pretherapeutic identification of high-risk tumors. Multiple methods have been developed to predict Ki-67 expression in HCC. For instance, Liu *et al*.^30^ demonstrated that T1 relaxation time measured via GD-EOB-DTPA-enhanced MRI T1WI mapping exhibits a strong positive correlation with HCC Ki-67 expression, offering excellent performance for non-invasive preoperative prediction of Ki-67 expression in HCC. Therefore, accurately assessing Ki-67 expression status holds critical clinical and oncologic significance for determining HCC biological behavior, predicting patient survival outcomes, and formulating individualized treatment plans (e.g., surgical extent, adjuvant therapy necessity, selection of targeted or immunotherapy drugs).^31,32^ However, traditional assessment methods rely on invasive biopsy, carrying risks of sampling error and posing challenges for dynamic monitoring of tumor proliferation, an important limitation in oncologic management. This study non-invasively predicted HCC Ki-67 expression status through T2WI-MRI habitat analysis, with results demonstrated that this model outperformed traditional whole-tumor radiomics models and clinical models based on serum AFP markers in both internal and external validation sets, providing a novel non-invasive imaging strategy for HCC molecular subtyping and personalized treatment that integrates oncologic principles into clinical practice.^33^

Compared to whole-tumor analysis, this method more effectively reveals functional subregions within tumors and their potential links to biological behavior.^34^ This advantage is particularly relevant to HCC oncogenesis, where intratumoral spatial heterogeneity drives treatment response and recurrence. This study employed k-means clustering (k = 5) to segment the T2WI images of HCC primary lesions into functional compartments. It successfully deciphered characteristic patterns of HCC heterogeneity and identified specific subregions at the tumor periphery significantly associated with high Ki-67 expression. These peripheral habitat subregions, enriched in proliferating cells, likely represent the “invasive front” of the tumor, which is a key oncologic feature that mediates local invasion and distant metastasis and is supported by the “tumor proliferation frontier” phenomenon. From these five habitat subregions, we further extracted high-throughput radiomics features. Through rigorous feature selection and machine learning processes, we constructed a predictive model for Ki-67 expression status. Key findings demonstrate that in an independent external validation cohort, this model exhibits high sensitivity and specificity, with predictive performance significantly superior to serum AFP levels (P < 0.001). Serum AFP is a conventional oncologic marker with limited specificity for assessing tumor proliferation. DCA indicated that applying this model yields a significant net benefit for patients when the clinical decision threshold probability exceeds 0.2, which underscores its clinical utility in oncologic decision-making. Furthermore, the model demonstrated excellent performance in the external validation dataset (AUC = 0.910, 95% CI: 0.8355–0.9835), confirming its high predictive accuracy for Ki-67 high expression status. In contrast, the predictive model combining serum markers and imaging features proposed by Cai *et al*.^35^ achieved AUC values of 0.854 and 0.833 on the training and internal validation datasets, respectively. However, internal validation alone is insufficient to fully validate the model’s general applicability in diverse oncologic cohorts. The habitat imaging model constructed in this study demonstrates high operational feasibility, reproducibility, and interpretability. It achieved high diagnostic performance in predicting HCC Ki-67 expression status using T2WI sequences, with a training set AUC of 0.984 (95% CI: 0.9647–1.0000). Through SHAP interpretability analysis, we further identified key habitat features driving model predictions, such as squareroot_glszm_ SmallAreaHighGrayLevelEmphasis_h2 (primarily derived from high-activity edge subregions; h2 is calculated from images processed by specific filters). These imaging features closely align with the “tumor proliferation frontier” phenomenon reported by Ye *et al*.^36^, highly enriched Ki-67-positive cells at the tumor-liver parenchyma interface, thus providing robust support for the model’s biological mechanism and highlights the oncologic implications of imaging habitats in guiding clinical management, risk stratification, and personalized oncologic therapy.

Despite promising results, we must cautiously consider the study’s limitations. First, this retrospective study draws data from a single regional medical center with a limited total sample size, particularly in the external validation set. This may introduce selection bias and constrain the model’s statistical efficacy and the generalizability of its findings. Future large-scale, multicenter prospective cohort studies are needed to further validate the robustness of our model. Second, despite manual tumor VOI segmentation by experienced radiologists, some degree of subjectivity remains unavoidable. We were unable to quantitatively assess intra- and inter-observer consistency, which requires refinement in subsequent work. Furthermore, this study developed the model based solely on T2WI sequences and has not yet explored the potential incremental value of integrating multi-parametric MRI (e.g., dynamic contrast-enhanced [DCE], DWI) habitat features. Potential impacts from varying MRI scanners and protocols were neither standardized nor evaluated within this study framework, which may challenge the model’s broad applicability. Third, our analysis focused solely on the Ki-67 proliferation marker, failing to compare or combine it with other critical proliferation-related markers (e.g., PCNA) or molecular subtypes. This limitation prevents a comprehensive assessment of the relationship between microenvironmental features and the overall biological behavior profile of HCC. Finally, current microenvironment partitioning algorithms primarily concentrate on the tumor entity itself, without systematically incorporating the heterogeneity of the cirrhotic background into the analytical framework. The potential interference of this background on partition stability and predictive efficacy warrants further investigation in future studies.

## Conclusions

In summary, our study confirms that habitat imaging based on conventional T2WI sequences, combined with k-means clustering (k = 5) to analyze HCC tumor microenvironment heterogeneity, enables highly accurate, non-invasive prediction of Ki-67 expression status. The constructed machine learning model demonstrates outstanding performance, clear biological significance, and remarkable clinical utility. This approach provides a powerful novel tool for preoperative assessment of HCC proliferative activity, overcoming limitations of traditional methods. The model can be implemented clinically using standardized T2WI sequences and common software, ensuring good generalizability. It holds promise as a key imaging biomarker in clinical practice to guide individualized treatment decisions, such as surgical extent, adjuvant therapy, and targeted/immunotherapy selection. Future research should focus on multicenter prospective cohort validation and explore integrating multimodal MRI information to further enhance predictive performance.

## Supplementary Material

Supplementary Material Details

## References

[1] Bray F, Laversanne M, Sung H, Ferlay J, Siegel RL, Soerjomataram I (2024). Global cancer statistics 2022: GLOBOCAN estimates of incidence and mortality worldwide for 36 cancers in 185 countries. CA Cancer J Clin.

[2] Yang M, Song X, Zhang F, Li M, Chang W, Wang Z (2025). Spatial proteomic landscape of primary and relapsed hepatocellular carcinoma reveals immune escape characteristics in early relapse. Hepatology.

[3] Jones A, Kroneman TN, Blahnik AJ, Graham RP, Mounajjed T, Torbenson MS (2021). Ki-67 “hot spot” digital analysis is useful in the distinction of hepatic adenomas and well-differentiated hepatocellular carcinomas. Virchows Arch.

[4] Sun H, Wang H, Wang Y, Zhong W, Meng Y, Lv Z (2023). Adjuvant transarterial chemoembolization timing after radical resection is an independent prognostic factor for patients with hepatocellular carcinoma. Front Oncol.

[5] Meng L, Jiang Z, Shen G, Lin S, Gao F, Guo X (2025). Genetic alterations are related to clinicopathological features and risk of recurrence/metastasis of hepatocellular carcinoma. Eur J Cancer Prev.

[6] Boukhar SA, Gosse MD, Bellizzi AM, Rajan KDA. (2021). Ki-67 proliferation index assessment in gastroenteropancreatic neuroendocrine tumors by digital image analysis with stringent case and hotspot level concordance requirements. Am J Clin Pathol.

[7] Lucas O, Ward S, Zaidi R, Bunkum A, Frankell AM, Moore DA (2025). Characterizing the evolutionary dynamics of cancer proliferation in singlecell clones with SPRINTER. Nat Genet.

[8] Jagtap SV. (2025). Evaluation and prediction of Ki-67 expression in hepatocellular carcinoma. World J Gastrointest Oncol.

[9] Xia TY, Zhou ZH, Meng XP, Zha JH, Yu Q, Wang WL (2023). Predicting microvascular invasion in hepatocellular carcinoma using CT-based radiomics model. Radiology.

[10] Liu HF, Wang M, Wang Q, Lu Y, Lu YJ, Sheng Y (2024). Multiparametric MRIbased intratumoral and peritumoral radiomics for predicting pathological differentiation of hepatocellular carcinoma. Insights Imaging.

[11] Huang Y, Chen L, Ding Q, Zhang H, Zhong Y, Zhang X (2024). CT-based radiomics for predicting pathological grade in hepatocellular carcinoma. Front Oncol.

[12] Romeo D, Richter T, Höhn AK, Tautenhahn HM, Seehofer D, Scheuermann U (2025). Associations between the SMARS score derived from CT and MRI with histopathological features in HCC. Sci Rep.

[13] Zhang Q, Lou Y, Bai XL, Liang TB. (2020). Intratumoral heterogeneity of hepatocellular carcinoma: from single-cell to population-based studies. World J Gastroenterol.

[14] Yang C, Zhang S, Cheng Z, Liu Z, Zhang L, Jiang K (2022). Multi-region sequencing with spatial information enables accurate heterogeneity estimation and risk stratification in liver cancer. Genome Med.

[15] Li H, Zhang J, Liu B, Zheng Z, Xu Y. (2025). Histogram analysis of multiple mathematical diffusion-weighted imaging models for preoperative prediction of Ki-67 expression in hepatocellular carcinoma. Front Oncol.

[16] Zhu Y, Wang J, Xue C, Zhai X, Xiao C, Lu T. (2025). Deep learning and habitat radiomics for the prediction of glioma pathology using multiparametric MRI: a multicenter study. Acad Radiol.

[17] Jhan SR, Wu YY, Chang PY, Chai JW, Su TC. (2023). Comparison of lesion detection ability of two MRI sequences of T2WI HASTE and T2WI BLADE for hepatocellular carcinoma. Medicine (Baltimore).

[18] Murakami K, Kasajima A, Kawagishi N, Ohuchi N, Sasano H. (2015). Microvessel density in hepatocellular carcinoma: prognostic significance and review of previous published work. Hepatol Res.

[19] Jones A, Kroneman TN, Blahnik AJ, Graham RP, Mounajjed T, Torbenson MS (2021). Ki-67 “hot spot” digital analysis is useful in the distinction of hepatic adenomas and well-differentiated hepatocellular carcinomas. Virchows Arch.

[20] Boukhar SA, Gosse MD, Bellizzi AM, Rajan KDA. (2021). Ki-67 proliferation index assessment in gastroenteropancreatic neuroendocrine tumors by digital image analysis with stringent case and hotspot level concordance requirements. Am J Clin Pathol.

[21] Nielsen TO, Leung SCY, Rimm DL, Dodson A, Acs B, Badve S (2021). Assessment of Ki67 in breast cancer: updated recommendations from the International Ki67 in Breast Cancer Working Group. J Natl Cancer Inst.

[22] Zhang W, Yue Z, Ye J, Xu H, Wang Y, Zhang X (2022). Modulation format identification using the Calinski-Harabasz index. Appl Opt.

[23] Wang A, He M, Zhang C, Zheng Y, Song Y, Wang C (2025). Intravoxel incoherent motion-based habitat imaging for predicting immunohistochemistry in patients with breast cancer. Front Oncol.

[24] Yang X, Niu W, Wu K, Li X, Hou H, Tan Y (2024). Diffusion kurtosis imagingbased habitat analysis identifies high-risk molecular subtypes and heterogeneity matching in diffuse gliomas. Ann Clin Transl Neurol.

[25] Ding Z, Zhang C, Xia C, Yao Q, Wei Y, Zhang X (2025). DCE-MRI-based deep learning analysis of intratumoral subregions for predicting Ki-67 expression level in breast cancer. Magn Reson Imaging.

[26] Maestri E, Kedei N, Khatib S, Forgues M, Ylaya K, Hewitt SM (2024). Spatial proximity of tumor-immune interactions predicts patient outcome in hepatocellular carcinoma. Hepatology.

[27] Lei HJ, Wang SY, Chau IY, Li AF, Chau YP, Hsia CY (2021). Hepatoma upregulated protein and Ki-67 expression in resectable hepatocellular carcinoma. J Chin Med Assoc.

[28] Li HH, Qi LN, Ma L, Chen ZS, Xiang BD, Li LQ. (2018). Effect of Ki-67 positive cellular index on prognosis after hepatectomy in BCLC stage A and B hepatocellular carcinoma with microvascular invasion. Onco Targets Ther.

[29] Ma J, Li D, Zhu X. (2024). Influencing factors for tumor thrombus in patients with hepatocellular carcinoma. Gastrointest Oncol.

[30] Liu Z, Yang S, Chen X, Luo C, Feng J, Chen H (2022). Nomogram development and validation to predict Ki-67 expression of hepatocellular carcinoma derived from Gd-EOB-DTPA-enhanced MRI combined with T1 mapping. Front Oncol.

[31] Ghamry HI. (2023). Impending chemotherapeutic impact of *Arthrospira platensis* nanoparticles and/or sorafenib against hepatocellular carcinoma through modulation of antioxidant status, tumor marker genes, and anti-inflammatory signaling pathways. Toxics.

[32] Yanai M, Kurata M, Muto Y, Iha H, Kanao T, Tatsuzawa A (2020). Clinicopathological and molecular analysis of SIRT7 in hepatocellular carcinoma. Pathology.

[33] Zhang X, Su GH, Chen Y, Gu YJ, You C. (2023). Decoding intratumoral heterogeneity: clinical potential of habitat imaging based on radiomics. Radiology.

[34] Tomaszewski MR, Gillies RJ. (2021). The biological meaning of radiomic features. Radiology.

[35] Cai C, Wang L, Tao L, Zhu H, Ren Y, Li D (2025). Imaging-based prediction of Ki-67 expression in hepatocellular carcinoma: a retrospective study. Cancer Med.

[36] Ye J, Lin Y, Liao Z, Gao X, Lu C, Lu L (2024). Single-cell spatial transcriptomics and bulk multi-omics analysis of heterogeneity and ecosystems in hepatocellular carcinoma. NPJ Precis Oncol.

