# Supplementary material for: Prediction of Ki-67 expression in hepatocellular carcinoma: a dual-center study based on T2-weighted imaging habitat analysis
Source: Radiol Oncol. 2026 Jun 26;60(2):217–26. doi: 10.2478/raon-2026-0032 (PMC13307014; doi:10.2478/raon-2026-0032)
Supplement: Supplementary file 1 — Supplementary Material Details [file raon-2026-0032_sm.pdf]

Prediction of Ki-67 expression in hepatocellular carcinoma: a dual-center study based on T2-weighted imaging habitat analysis

Xiaojun Zheng, Lihong Huang, Mengjie Huang, Bin Yu, Shiji Qin, Deyou Huang

doi: 10.2478/raon-2026-0032

SUPPLEMENTARY TABLE 1. Scanning parameters for different MRI scanners

Equipment	TR/TE (mm/ms)	FOV (mm)	Flip angle	Thickness (mm)	Matrix
1.5T Siemens Amira	2500/80	420 × 420	170	6	320×320
1.5T Siemens SIGNA Creator	4444/82	400 × 400	160	6	320×320
3.0T GE Discovery MR 750	6667/82	360 × 360	112	6	320×320
1.5T Siemens MAGNETOM Skyra	3000/79	380 × 380	180	6	320×320
1.5T Canon Vantage Elan MRF 2020	6524/90	370 ×410	160	7	256×320

FOV = field of view; TE =echo time; TR = repetition time
